# Femtosecond laser cataract surgery

**DOI:** 10.1186/s40662-015-0021-7

**Published:** 2015-06-30

**Authors:** Zoltan Z. Nagy, Colm McAlinden

**Affiliations:** Department of Ophthalmology, Semmelweis University, Maria u. 39, H-1085 Budapest, Hungary; Flinders University, Bedford Park, Adelaide, South Australia Australia; School of Ophthalmology and Optometry, Wenzhou Medical University, Wenzhou, Zhejiang China

**Keywords:** Femtosecond, Cataract surgery, Femtosecond laser, Capsulorhexis, Phacoemulsification, Corneal incisions, Corneal oedema, Macular oedema

## Abstract

Femtosecond laser (FSL) cataract surgery is in its infancy but is rapidly gaining popularity due to the improved consistency and predictability for corneal incisions and anterior capsulorhexis. It enables subsequently less phacoemulsification energy and time to be employed, which has gains in terms of reduced corneal oedema. In addition, the FSL allows better circularity of the anterior capsulotomy, capsule overlap, intraocular lens (IOL) placement and centration of the IOL. These advantages have resulted in improved visual and refractive outcomes in the short term. Complication rates are low which reduce with surgeon experience. This review article focuses on the Alcon LenSx system.

## Introduction

**Fig. 1:**
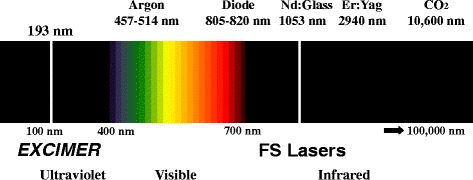
Lasers in ophthalmology; excimer lasers operate in the ultraviolet range whereas femtosecond lasers operate in the infrared range of the electromagnetic spectrum

Ophthalmology has always been at the forefront in the use of lasers (Light Amplification by Stimulated Emission of Radiation) and a variety of lasers have been employed for more than 50 years [1–3]. In 1949, German ophthalmologist Dr. Gerhard Meyer-Schwickerath suggested photocoagulation of the retina with lasers [4]. A laser is a device that emits electromagnetic light via stimulated emission. Ophthalmic lasers operate at one specific fixed wavelength, pulse pattern, energy, duration, repetition rate, and spot size. This enables photons in phase from the coherent, monochromatic laser beam to arrive at the same time and place in the target tissue. Hence, by modifying these parameters they may be absorbed in various tissues at various depths with varying biological effects. This has resulted in lasers being able to target any issue within the eye. Many lasers operate by molecular vibration, which causes localized thermal effects such as photocoagulation (e.g. argon laser) [5] resulting in protein denaturation. Other lasers operate by photoablation such as the excimer laser used in refractive surgery [6, 7] and by photodisruption such as the neodymium: yttrium-aluminium-garent (Nd:YAG) laser [8] (Fig. 1).

The Nd:YAG laser has a pulse duration in the nanosecond range (10^−9^) and causes photodisruption at its focal point, such as the posterior capsule of the lens. However, the Nd:YAG laser causes collateral damage and is the reason why it is not used in corneal procedures. However, by shortening the wavelength further to the femtosecond range (10^−15^), there is a reduction in acoustic shock waves that in turn results in markedly less collateral damage. This also results in a more precise tissue effect [9, 10].

**Fig. 2:**
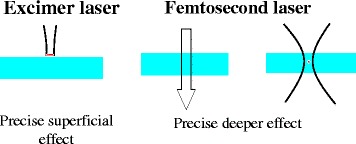
Corneal surgery based on photodisruption. Excimer lasers produce precise superficial effects whereas femtosecond lasers produce precise deeper effects within the cornea and the crystalline lens

The femtosecond laser (FSL) first appeared in corneal refractive surgery as a microkeratome to create the corneal flap during laser in situ keratomileusis (LASIK) [11]. Thereafter the indication has expanded to all types of corneal surgery including lamellar and penetrating keratoplasty, ring-segment implantation in keratoconus and presbyopia inlay pocket creation [12, 13]. The first corneal FSLs operated with 30 kilohertz, (kHz)). The repetition rate was then doubled to 60 kHz and the latest 160 kHz FSLs are able to create a corneal flap within 10 s. With higher repetition rates, less energy is required to obtain the same tissue effect. FSLs used in cataract surgery employ a pulse duration of 400–800 femtoseconds (fs) and the energy range is in microjoules (μJ, 10^−6^ J). During the surgery of the crystalline lens of the eye, the FSL energy may be increased to 10–15 μJ (Fig. 2).

The photodisruptive effect is achieved when the FSL beam is sharply focused and generates plasma within the affected tissue. This plasma rapidly expands as an acoustic shock wave, displacing the surrounding tissue. With time, the plasma cools and cavitation bubbles are formed. At tissue level, photodisruption occurs at the laser’s focal point without any thermal effect or collateral tissue damage [14]. With this photodisruptive effect, the FSL is capable of creating tissue separation and very precise cuts within the cornea, lens capsule and crystalline lens.

One of the most important characteristics of the FSL during corneal and lens use is the numerical aperture. The numerical aperture significantly affects both the spot size and volume. With a larger numerical aperture, less dispersion of the laser beam ensues that in turn provides a better focused laser beam. Further, the precision of the cut depth improves and lower energy is needed to provide the same tissue effect. Therefore, corneal treatments require a larger numerical aperture (with a lower energy level), and vice versa with the crystalline lens. It is important that a FSL treating both the cornea and the crystalline lens simultaneously should have adequate flexibility in the energy, pattern and duration of the pulse as well as the repetition rate [15].

Cataract surgery is the most commonly performed ophthalmic procedure worldwide [16, 17]. Cataract surgery has shifted its focus from a purely vision restoration procedure to a refractive procedure. Ophthalmic surgeons not only restore clarity of the optical media, but also change the refractive power of the eye. Further, presbyopic treatment is now possible with the use of multifocal intraocular lenses (IOLs) [18].

The accuracy of corneal laser refractive surgery is significantly higher than in cataract surgery [19], therefore much work is needed to improve refractive outcomes. Patient expectation has risen and doctors need to be proficient at communicating with patients to manage expectations accordingly [20]. FSLs offer new potential for patients and surgeons alike.

## Review

### Technical aspects of FSLs

**Fig. 3:**
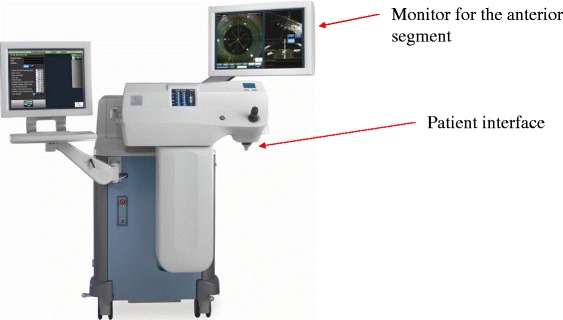
The LenSx femtosecond laser

The Alcon LenSx FSL operates with a solid state laser source that produces thousands of fs pulses per second (Fig. 3). The laser pulses are delivered via a sophisticated beam delivery system. This system includes an articulated arm, a series of optical lenses, scanners and monitors. The Alcon LenSx laser system is capable of providing a variable numerical aperture for optimal performance in both the corneal and lens plane [15].

The first part of FSL assisted cataract surgery with the Alcon LenSx is the docking procedure. The surgeon uses a curved contact lens, which is integrated with a sterile limbal suction ring (SoftFit PI). The tubing uses a vacuum system to fixate the eye. The patient interface is simple to dock and it provides a large viewing and surgical diameter range that allows the surgeon to perform the peripheral corneal incisions and arcuate keratotomy incisions. The patient interface is associated with elevations in intraocular pressure (IOP) of between 16 and 20 mmHg, which supports conservation of vision and ocular perfusion during the FSL pretreatment [21].

**Fig. 4:**
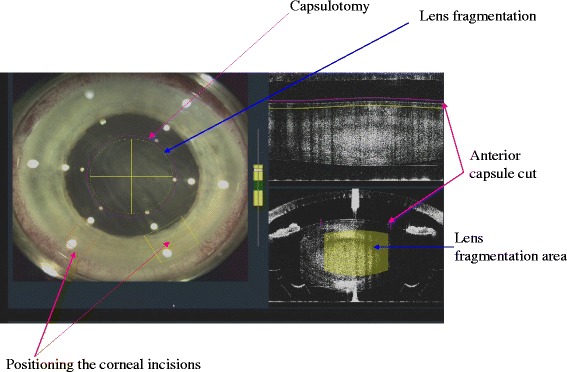
The optical coherence tomography (OCT) system provides information for the surgeon and the surgical plan is based on the pre-operative OCT measurements (size and centration of capsulotomy, fragmentation pattern, corneal wound structure and geometry, and arcuate astigmatic incisions)

The Alcon LenSx system has a built-in high definition optical coherence tomography (OCT) system and a live video to assist with the docking and surgical pattern localization (Fig. 4). The OCT uses the same optical path as the laser beam and is fully integrated and calibrated. The OCT covers the complete anterior segment of the eye up to the posterior capsule of the crystalline lens. It is also capable of assessing the lens density. The LenSx automatically performs the surgical pattern after preoperative planning by the surgeon and the selection of settings. The surgeon is able to alter the various treatment parameters, such as the corneal incision positions, centration of the anterior capsulotomy, and cut depth within the crystalline lens.

With the new software version and coupling by the Verion pre-operative assessment system, conjunctival, scleral vessels and iris characteristics are identified and recognised. This provides automated recognition and centration of the procedure.

The first human FSL assisted cataract surgery was performed with the Alcon LenSx in 2008 by Zoltan Nagy at Semmelweis University in Budapest, Hungary [22]. Since then the United States Food and Drug Administration (FDA) has granted approval and the European Conformité Européene (CE) mark has been granted for FSLs, and FSLs are available for the public. There are currently four international companies producing and providing this new technology for cataract surgery. Peer-reviewed publications are increasing on the subject and it is predicted that within a decade, the method will be available in most of the large ophthalmic centres around the world [23].

### Indications

The main indications of FSL during cataract surgery are:

- Anterior capsulotomy creation
- Laser fragmentation and liquefaction of hard and soft lenses respectively
- Single plane or multiplane corneal incisions
- Arcuate corneal incisions to control pre-operative corneal astigmatism

The diameter of the anterior capsulotomy can be varied between 4.5 and 6 mm with the LenSx system. For lens fragmentation, a hybrid pattern may be used. This involves liquefaction of the central 3.0 mm core and fragmentation of the peripheral lens in 4 to 8 cuts (cross pattern and cake or pizza pattern). This method enables the removal of the central lens providing access to the remaining lens periphery. This therefore reduces the ultrasonic phacoemulsification energy and time [24, 25] to provide better one day post-operative visual acuity, decreased corneal and cystoid macular oedema (CMO). Other advantages include reduced manipulation within the eye due to the pre-fragmented and liquefied lens. Most studies have limited treatments with the FSL up to grade 4 on the nuclear scale on the Lens Opacities Classification System (LOCS) [26]. In advanced brunescent cataracts, the water content of the lens is low therefore, there is less absorption of the laser. With white tumescent cataracts, the water content is high hence, efficient lens fragmentation is very unlikely. In such cases, the FSL is limited to the anterior capsulotomy and corneal incisions.

### Contraindications

- Corneal scarring
- Mature cataract (relative contraindication)
- Small, non-dilating pupil (relative contraindication)

The small, non-dilating pupil less than 6 mm in diameter is recognised as a relative contraindication to FSL cataract surgery (lens fragmentation). It is possible to perform an anterior capsulotomy with a 5.0 mm pupil, but there is a high risk of iris injury. Malyugin rings or iris hooks may be used in such cases [27]. Corneal incisions can still be performed in cases of small pupils.

### Ordering of steps

The order of performing the three main steps is as follows: first, the anterior capsulotomy is created; second, the lens fragmentation and or liquefaction; and third, the corneal incisions. The anterior capsulotomy is performed before the lens fragmentation/liquefaction because the lens fragmentation/liquefaction may create a gas bubble that may elevate the anterior capsule. The corneal incisions are performed last as they are performed from the inside to outside.

### Clinical results

#### Anterior capsulotomy

Initial ex vivo evaluation of the accuracy of the anterior capsulotomy, targeting for 5 mm found that using the standard manual technique, the diameter was 5.88 (± 0.73) but 5.02 (±0.04) mm using the Alcon LenSx FSL. In human eyes, the Alcon LenSx FSL achieved all capsulotomies within ± 0.25 mm whereas with the manual technique it was only achieved in 10 % of the eyes [22].

The capsulorhexis is crucial to achieve an accurate final post-operative refraction. As the manual technique has been the only method available, little research has focused on the effect on the refractive outcome. A larger than intended rhexis may cause anterior or posterior shift of the IOL or IOL tilt and also can increase the risks of posterior capsular opacification. A capsule strength study found that capsulotomies created with the OptiMedica FSL required two to three times more force to tear compared with the manual capsulotomies [28]. The effective lens position (ELP) is a very important parameter in IOL calculation especially with multifocal IOLs [29, 30] and the accuracy of the size and position of rhexis is thus important in this regard [31, 32]. In a study by Dick and Schultz using the Catalys Precision Laser System, they concluded that the final ELP position can be achieved sooner and may allow more predictable refractive outcomes [33]. A recent study by Packer et al. reported that constructing the capsulotomy centred on the clinical approximation of the optical axis of the lens with a diameter of 5.25 mm optimizes the consistency of the ELP. On the contrary, manual capsulorhexis studies have not consistently shown an effect of size and centration on postoperative ELP and possible reasons are discussed by Packer et al. [34].

#### Anterior capsulotomy circularity and PCL centration

**Fig. 5:**
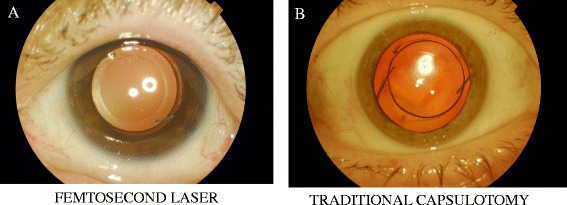
Femtosecond laser created capsulotomy versus a traditional manually created capsulotomy

Authors performed two studies at Semmelweis University in Budapest in an attempt to ascertain the accuracy of the circularity of the FSL rhexis with the Alcon LenSx and the effect on IOL centration postoperatively. They found that the LenSx performed anterior capsulotomy was more regularly shaped and provided better centration and capsule/IOL overlap compared with the manual capsulorhexis (Fig. 5). Vertical and horizontal IOL decentration following the manually created rhexis was statistically higher than the LenSx [31, 35].

In another anterior capsulotomy comparative study, the Alcon LenSx FSL was used to create an anterior capsulotomy in 54 eyes and the standard manual technique used in 57 eyes. The circularity was statistically significantly better in the FSL group (*p* = 0.032) and there was significantly less incomplete overlap of capsulotomies with the manual rhexis (28 % of eyes versus 11 %; *p* = 0.033) [36].

#### Lens fragmentation and phacoemulsification energy

The various FSLs offer different types of lens fragmentation. With soft lenses, LOCS grading less than 2.0, a central 5 mm central liquefaction is typically used, creating concentric rings (cylindrical pattern) within the nucleus of the crystalline lens. This scenario is more likely seen in younger patients considering refractive lens exchange. With LOCS grading greater than 2.0, lens fragmentation is typically used. This can be a cross pattern (2 perpendicular incisions within the lens), or can be customized with an increased number of cuts. On the LenSx, they are termed cake or pizza pattern fragmentation providing 6 to 8 cuts.

With the LenSx, the cubicle pattern may also be used, but two main planes are still required in order to chop the crystalline lens and to avoid the epinuclear ‘bowl’ that can be difficult to remove either with the phaco hand-piece or during irrigation-aspiration [25].

The hybrid pattern on the LenSx may be used with a central 3 mm diameter liquefaction with peripheral lens fragmentation lines. With this method, the surgeon is able to reduce the required phaco energy and time further, thus increasing the safety of the method. The liquefaction and fragmentation diameter area should not be greater than 1 mm of the capsulorhexis diameter due to the concave shape of the back of the lens surface. With longer fragmentation lines, one increases the risk of trauma to the posterior capsule. A study by Takács et al. compared FSL with the LenSx to non-FSL cataract surgery finding the mean phaco energy of 20.4(±12.6)% in the non-FSL group compared to 12.7(±8.3)% in the FSL group which was statistically significant (*p* < 0.05). Corneal endothelial cell counts were slightly lower in the non-FSL group at all postoperative visits but differences were not statistically significant [37].

The use of the cross pattern and ‘quick chop’ mode with the LenSx FSL compared to standard phacoemulsification resulted in a 43 % reduction in cumulative dissipative energy and a 51 % reduction in phacoemulsification time using the Infiniti (Alcon, Forth Worth, Texas, USA) phacoemulsification device [22].

#### Corneal and limbal incisions

Manually created incisions with a blade may have imprecise tunnel structure and length, which usually require stromal hydration at the end of surgery. At low IOP, the manual wound might become unstable, permitting bacterial entry from the conjunctival sac, leading to endophthalmitis [38]. Corneal wounds with precise geometry and architecture are of utmost importance in controlling postoperative infection and minimising surgically induced astigmatism [39]. Therefore, the capabilities offered by the FSL, such as precise wound geometry and architecture with more consistency, deliver better wound seal without the need for stromal hydration at the end of the procedure [40, 41]. Wound characteristics are also important for IOL selection, especially toric and multifocal IOLs [42]. Incisions can be corneal or limbal relaxing and case reports with the LenSx and Catalys OptiMedica FSLs indicate successful correction of large amounts of corneal astigmatism [43, 44].

The LenSx uses an image-guided capability enabling the control of corneal thickness, incision length, width, depth, shape, and location. The procedure is computer-controlled, precise and predictable. It is possible to open the incision immediately following the FSL pre-treatment or wait until the next post-operative day. With the aid of topography, this may be used to adjust the surgically induced astigmatism at the slit lamp.

#### Refractive outcomes

A study from Semmelweis University, Budapest, compared internal aberrations and quality of vision in eyes treated with the LenSx FSL and standard manual phacoemulsification. The study found that the anterior capsulotomy with the LenSx induced significantly less internal aberrations as measured by the Optical Path Difference (OPD) scanner (NIDEK Inc, Japan) [45]. Other outcome comparisons included: post-operative visual acuity (uncorrected and best corrected spectacle), residual refraction, ocular and internal aberrations, Strehl ratio and the modulation transfer function (MTF). No statistically significant differences were found between the post-operative refraction and distant visual acuity (uncorrected and corrected). At all measured cycles per degree, the femtosecond treated eyes had lower values of intraocular vertical tilt (Z_1_^−1^) and coma aberrations (Z_3_^−1^), higher Strehl ratios, and higher MTF values (*p* < 0.05) [45]. Other studies in the literature have reported no difference in postoperative refractive error between FSL and standard cataract surgery [46–50]. However, Szigeti and colleagues found reduced IOL tilt and decentration with a FSL created anterior capsulorhexis of 5.5 mm compared with 6 mm [48]. Subjective comparative outcomes between FSL cataract surgery and standard cataract surgery in terms of quality of vision [51–53] and quality of life [54–57] are welcomed.

#### Effects on the corneal endothelium

It is estimated that there is an 8.5 % endothelial loss associated with standard cataract surgery [37]. Abell and colleagues found no difference in endothelial cell loss between the Catalys OptiMedica FSL and standard cataract surgery three weeks post-operatively [58]. Abell performed FSL cataract surgery in a group of 405 eyes where 118 had automated laser corneal incisions and 287 had manual corneal incisions. Endothelial cell loss was significantly less in eyes with manually created corneal incisions [59]. Takács and colleagues compared the corneal thickness, corneal volume stress index, and endothelial density following FSL with the LenSx and standard cataract surgery. They reported better outcomes with the LenSx in the early post-operative period in terms of significantly lower corneal thickness compared to the manual group; and the difference disappeared after one week and one month. Purported explanations for this finding include the reduced phacoemulsification time and corneal oedema associated with the FSL technique [37].

#### Effects on the macula

In a study comparing changes in macular thickness following FSL with the LenSx versus standard cataract surgery, there were less gains in macular thickness in the FSL group at one week post-operatively. There was no difference after one month [60]. Nagy et al. also compared thickness changes in the retinal layers in the macula with OCT after femtosecond laser-assisted phacoemulsification (study group) and conventional phacoemulsification (control group). After cataract surgery, macular oedema was detectable mainly in the outer nuclear layer in both groups but was significantly less using the femtosecond laser platform [61]. However a recent randomised controlled trial of 102 patients by Conrad-Hengerer et al. found no significant difference in the occurrence of macular oedema between FSL cataract surgery and standard phacoemulsification [62]. Further, Levitz et al. recently reported no difference in macular oedema between groups of patients undergoing FSL cataract surgery compared to standard phacoemulsification [63].

### Complications

Complications are rare with the FSL. The most common issues encountered are:

#### Pupillary constriction

The pupil diameter should be at least 6 mm to avoid injury to the iris. Shock waves from the FSL can be close to the iris thereby causing inadvertent miosis. Additional dilating drops and non-steroidal anti-inflammatory drugs (NSAIDS) may be used pre-operatively in cases of small pupils.

#### Capsular blockage syndrome

Roberts and colleagues were the first to report capsular block syndrome, occurring with the LenSx [64]. Capsular block syndrome may arise during hydrodissection whereby high speed fluid ingress can impede the gas bubble to escape the nucleus that increases intra-lens pressure and subsequent posterior capsular rupture followed by a dropped nucleus into the vitreous cavity. The ‘rock and roll’ technique described by Nagy reduces the risk of this possible complication [65]. The technique involves titred injection of hydrodissection fluid and careful splitting of the nucleus that helps to release intra-lenticular gas bubbles. The rock refers to gentle downward force to the lens and the roll refers to the gentle moving of the lens.

#### Corneal incisions

During the planning stage pre-operatively with the LenSx, surgeons should ensure proper centration of the patient interface to avoid incorrect corneal incision placement such as more central than expected, which may induce corneal astigmatism. Further precise docking is imperative to avoid lens tilt, shifted corneal incisions, which in turn may lead to asymmetrical or incomplete capsulotomy creation and lens fragmentation [65].

Other complications reported in the literature include anterior capsule tear (with the Catalys) [66], corneal perforation (with the Catalys) [67] and large increases in IOP (with the Victus platform) [68]. In a large study by Abell et al. involving 4080 eyes, with 1852 having had FSL cataract surgery with the Catalys Precision Laser System, an incomplete capsulotomy was found to occur in 21 eyes. An anterior capsulotomy tag occurred in 30 eyes compared to 1 with non-FSL surgery (*p* = 0.001). Posterior capsular tear occurred in 8 eyes compared to 4 with non-FSL surgery (not statistically significant). Corneal haze occurred in 12 eyes compared to 1 with non-FSL surgery (*p* = 0.0009). An unstable pupil occurred in 30 eyes compared to 14 with non-FSL surgery (*p* = 0.003) [66].

### Special indications

FSL cataract surgery has been successfully used in ocular trauma [69], phacomorphic glaucoma [27], penetrating keratoplasty [70], keratoconus, Marfan syndrome [71], in a nanophthalmic eye [72], and paediatric cataract surgery [73]. The interested reader is directed to the cited papers for further reading.

## Conclusion

The FSL is utilized in three main areas: corneal incisions, anterior capsulotomy, and lens fragmentation. Aside from improved precision and accuracy, the subsequent energy requirements for phacoemulsification as well as the phacoemulsification time are significantly less. The FSL is not in widespread use at present possibly due to the additional cost with limited gains over an already effective procedure.

New developments are expected, such as phacoemulsification tips and smaller incision options. Combined FSL and phacoemulsification machines that simultaneously treat the lens and the cornea are anticipated.
